# Evaluation of coat color inheritance and production performance for crossbreed from Chinese indigenous Chenghua pig crossbred with Berkshire

**DOI:** 10.5713/ab.21.0574

**Published:** 2022-03-03

**Authors:** Yujing Li, Rong Yuan, Zhengyin Gong, Qin Zou, Yifei Wang, Guoqing Tang, Li Zhu, Xuewei Li, Yanzhi Jiang

**Affiliations:** 1 Department of Zoology, College of Life Science, Sichuan Agricultural University, Ya’an 625014, Sichuan, China; 2 Chengdu Livestock and Poultry Genetic Resources Protection Center, Chengdu 610081, Sichuan, China; 3 Institute of Animal Genetics and Breeding, College of Animal Science and Technology, Sichuan Agricultural University, Chengdu 611130, Sichuan, China

**Keywords:** Berkshire, Chenghua Pig, Coat Color, Crossbred, Melanocortin 1 Receptor (*MC1R*), Production Performance

## Abstract

**Objective:**

This work was to determine coat inheritance and evaluate production performance for crossbred pigs from Berkshire×Chenghua (BC) compared with Chinese indigenous Chenghua (CH) pigs.

**Methods:**

The coat color phenotypes were recorded for more than 16,000 pigs, and the genotypes of melanocortin 1 receptor (*MCIR*) gene were identified by sequencing. The reproductive performance of 927 crossbred BC F4 gilts and 320 purebred CH gilts was recorded. Sixty pigs of each breed were randomly selected at approximately 60 days of age to determine growth performance during fattening period, which lasted for 150 days for BC pigs and 240 days for CH pigs. At the end of the fattening period, 30 pigs of each breed were slaughtered to determine carcass composition and meat quality.

**Results:**

The coat color of BC pigs exhibits a “dominant black” hereditary pattern, and all piglets derived from boars or sows genotyped *E^D1^E^D1^* homozygous for *MC1R* gene showed a uniform black coat phenotype. The BC F4 gilts displayed a good reproductive performance, showing a higher litter and tear size and were heavier at farrowing litter and at weaning litter than the CH gilts, but they reached puberty later than the CH gilts. BC F4 pigs exhibited improved growth and carcass characteristics with a higher average daily live weight gain, lower feed-to-gain ratio, and higher carcass lean meat rate than CH pigs. Like CH pigs, BC F4 pigs produced superior meat-quality characteristics, showing ideal pH and meat-color values, high intramuscular fat content and water-holding capacity, and acceptable muscle-fiber parameters. C18:1, C16:0, C18:0, and C18:2 were the main fatty acids in *M. longissimus lumborum* in the two breeds, and a remarkably high polyunsaturated/saturated fatty acid ratio of ~0.39 was observed in the BC F4 pigs.

**Conclusion:**

The BC F4 pigs exhibit a uniform black coat pattern and acceptable total production performance.

## INTRODUCTION

The Chenghua (CH) pig is a traditional black breed native to southwestern China in Sichuan Province, and it is characterized by superior meat quality characteristics and good adaptability to extensive management [1]. However, due to undesirable attributes such as slow growth rate and low lean meat percentage [2], the production system of purebred CH pigs has been almost displaced, while CH pigs have been included in the National Program for Farm Animal Resources since 2014.

Crossbreeding programs have been used extensively to improve the native pig’s overall production performance while maintaining superior meat quality for F1 hybrid pigs from Duroc×Dahe, Duroc or Landrace×Celta, Duroc×Korean Native Black Pig, and Duroc× Berkshire (BK)×Yanan [3–6]. However, due to the break-up of epistatic complexes since the F2 generation [7,8], improving and stabilizing the obtained heterosis based on breed additive and dominance effects is a considerable challenge for new breed formation arising from the crossing of two or more existing breeds.

In recent years, to utilize the genetic resource of the CH pig to improve its overall production performance and produce superior meat, we have implemented the crossbreeding scheme of BK×CH (BC) and bred the new crossbred BC pig through selection for four generations. Currently, the core breeding group of BC pigs contains 30 unrelated boar strains and more than 1,000 sows, and the production system of BC pigs can supply approximately 50,000 black fattening pigs per year to meet the market demand for high quality pork. However, scientific data evaluating the production performance and coat color variation for the new crossbred BC pigs is lacking. Therefore, the objective of this work was to determine the black coat inheritance and evaluate production performance for crossbred BC F4 pigs in comparison with the same assessments of control, purebred CH pigs.

## MATERIALS AND METHODS

All animal experimental procedures were approved by the Instituional Animal Care and Use Committee of Sichuan Agricultural University (permit number: SKY-2021216012).

### Breeding group structure and management

All pigs were maintained on the Qionglai Jialin Ecological Farm, Chengdu city, China. The core breeding group of purebred CH pigs included 8 unrelated boar strains and 320 sows. The BC crossbred base population derived from the progeny of 10 BK unrelated boar strains and 218 CH sows with the above sow selection index (SSI). The more advanced generations were bred by the method of population subgeneration breeding, and the crossbreeding population included 30 unrelated boar strains and approximately 600 sows in each generation. Mating was performed in a way that reduced inbreeding. Animals with a relationship coefficient above 5% were not mated with each other. All mattings were performed through artificial insemination. The selection of boars and gilts was based mainly on the boar selection index (BSI) and SSI, respectively; meanwhile, the selection was also in combination with pedigree and phenotypic characteristics. The management and feeding conditions of all pigs at different stages of production were largely designed according to the conditions that are experienced in modern breeding areas. The diets met the National Research Council (NRC) [9] recommendations for the different production phases.

The model fitted for the boar and SSI were:


$$\begin{array}{l} BSI=100+8.25{EBV}_{WT200}-4.78{EBV}_{BF200}\\ SSI=100+20.35{EBV}_{NBT}+4.62{EBV}_{WT200}-2.56{EBV}_{BF200} \end{array}$$

where *BSI* is the boar selection index while *SSI* is the sow selection index; *EBV_WT200_* and *EBV_BF200_* are the estimated breeding values for live weight and backfat thickness at 200 days of age, respectively; *EBV_NBT_* is the total number of pigs born per litter.

### Observation of coat color variation and collection of reproductive performance data

Coat color was observed for cross piglets per litter and their parents, and “the uniform black” or “domino black spotting” phenotypes were recorded for more than 16,000 pigs. The reproductive performance of 927 crossbred BC F4 gilts and 320 purebred CH gilts was recorded and collected from January 2019 to July 2020. The number of teats was recorded for gilts, and the puberty of gilts was defined as the first observed estrus followed by a second estrus approximately 21 or 42 d later. The total number of pigs born and number of pigs born alive per litter were recorded, while piglets per litter were weighed within 12 h of birth and at 28 d of age for litter weight at birth and litter weight at weaning, respectively.

### Identification of MC1R single nucleotide polymorphisms

Hairs with follicles were collected after disinfection with 75% alcohol at the shoulder, washed twice with phosphate-buffered saline and stored in a refrigerator at −20°C. Genomic DNA was extracted using a DNA extraction kit according to the manufacturer’s instructions (Magen, Guangzhou, China). Two pairs of primers were designed according to the melanocortin 1 receptor (*MC1R*) reference sequence (GenBank accession number FJ6655467.1) to amplify the complete MC1R DNA sequence (Table 1). The polymerase chain reaction (PCR) system was 25 μL, containing 22 μL 2×TsingKE Master Mix (TsingKE, Beijing, China), 1 μL upstream primer, 1 μL downstream primer, and 1 μL DNA. Thermocycling conditions began with denaturing at 98°C for 2 min, followed by 34 cycles of denaturing at 98°C for 10 s, annealing at the Tm (Table 1) for 10 s, extension at 72°C for 10 s, and finally extension at 72°C for 5 min. The samples were stored at 4°C. The amplification process was conducted using a Genemate Series PCR machine (Analytik Jena, Jena, Germany).

An aliquot of 5 μL of PCR product was used for 1.5% agarose gel electrophoresis to determine whether the *MC1R* gene was amplified. BigDye Terminator V3.1 was used for sequencing purification. A 3,730 sequencer was used for sequencing, and 3730XL was used for data collection. The sequencing sequence and peak graph were obtained by Chromas. The obtained sequence was spliced by CExpress to obtain a complete sequence. The sequence was aligned by BLAST in NCBI.

### Measurement of fattening and slaughtering performance

In total, 120 pigs (30 castrated males and 30 females for BC F4 or CH pigs, respectively) were randomly selected at approximately 60 days of age (with weight at approximately 15 kg). These pigs of each breed were born to a total of 15 litters that were produced by five sires and 15 sows. All pigs were housed in individual pens (2 m^2^) located in the same room and were fed twice a day with the same diet, and pigs had *ad libitum* access to diet and water (nipple drinkers). For the pigs to gain the expected market slaughter weight and age, the fattening experiment lasted for 150 days for BC pigs and 240 days for CH pigs after the 7 days adaptation period. The experimental diets met the National Research Council (NRC) [9] recommendations for the two different growth phases. In the fattening period, the data of initial live weight, final live weight and feed consumption were recorded to determine daily live weight gain and feed-to-gain ratio.

At the end of the fattening period, 30 pigs (15 castrated males and 15 females) of each breed were slaughtered to determine carcass-composition characteristics according to the described methods [3,6]. The measure carcass attributes included carcass length, dressing percentage, back fat thickness, loin muscle area, skin thickness, number of ribs, and dissection ratio of bone, muscle, subcutaneous fat and skin. The *M. longissimus lumborum* of the left side of the carcass at the last third to fourth rib was sampled and used to measure meat quality according to the described methods [3,6]. The measure meat quality properties included pH values, color parameters, water-holding capacity, and muscle fiber parameters. The muscular fatty acid (FA) composition was analyzed using gas chromatography (Agilent 6820, Agilent Technologies, Palo Alto, CA, USA) and capillary column (HP-Innowax, Agilent, 30 m long, 0.32 mm internal diameter, 0.25 mm film thickness) according to the described method [10].

### Statistical analyses

Statistical testing was implemented by IBM SPSS Statistics 22. The data are quantified as the mean±standard error of the mean for one group. The differences between groups were calculated using an independent T test. Statistical significance is defined when p values are less than 0.05, * p<0.05, ** p<0.01, and *** p<0.001.

## RESULTS

### Specific black coat were selected for the crossbred BC pigs based on the *E^D1^E^D1^* homozygous genotype of the *MC1R* gene

**Figure 1:**
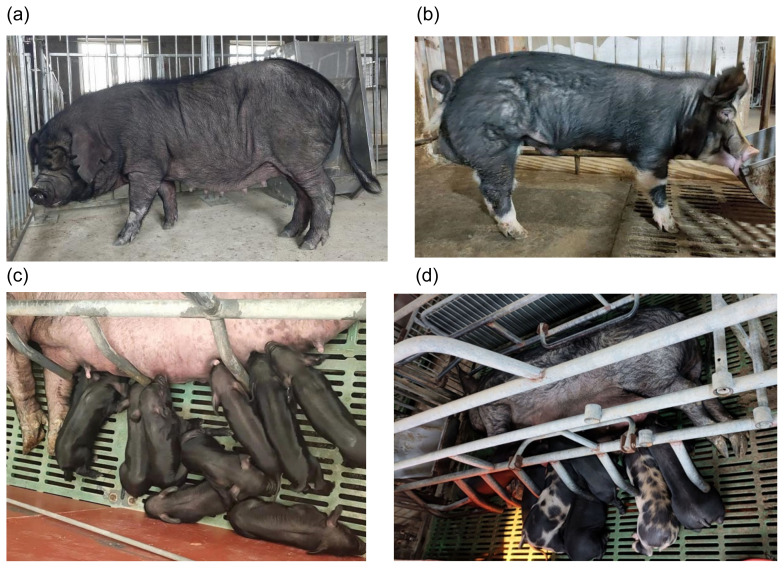
Phenotypic characteristics of hair color in different pig populations. (a) uniform black type for pure Chenghua pig; (b) domino black spotting type for Berkshire pig; (c) uniform black type for the cross F1 from Berkshire×Chenghua (BC); (d) Color separation with black type and “black-white” type for the cross F2 from Berkshire×Chenghua (BC).

To identify the hereditary pattern of black coat for the crossbred BC pigs, we first observed the phenotypic changes of coat color in cross generation. As a result, all F1 crosses (3,140), which were derived from 10 BK boars (domino black spotting type) and 320 CH sows (black type), were uniform black. However, F2 cross pigs (5,906), which were derived from 30 F1 black boars and 588 F1 black sows, were black or domino in the proportion of approximately 3:1 (Figure 1). Interestingly, when 26 F2 black boars were used in the production system, F3 cross pigs (2, 038 out of 2,041) derived from 9 F2 black boars (called homozygote) and 182 F2 black sows were uniform black, but F3 cross pigs (3,698) derived from the other 17 F2 black boars (called heterozygote) were black or domino in the proportion of approximately 5:1 (Table 2). The results indicated that the black coat of crossbred BC pigs might be controlled by a dominant single gene and could be inherited in accordance with Mendel’s law of segregation.

Based on the important regulatory role of *MCIR* gene on body melanin deposition [11], we considered the *MC1R* gene as a potential candidate gene for the black coat of crossbred BC pigs and cloned and sequenced the complete DNA of the *MC1R* gene for these samples from BK, CH, F1 crosses, F2 black boars (homozygous or heterozygous), and F2 domino black spotting cross pigs. As a result, we obtained a 1,552 bp DNA sequence of MC1R (GenBank accession number AY 960624) and screened 12 mutation sites in the complete DNA sequence of the *MC1R* gene from these samples (Table 3). According to the definitely established alleles at the MC1R locus [11], we found that the CH pigs and F2 black boars (homozygotes) showed the typical *E^D1^E^D1^* homozygous genotype, while BS and F2 crosses with domino black spotting showed other opposite *E^P^E^P^* homozygous genotypes; meanwhile, F1 crosses and F2 black boars (heterozygotes) displayed the same *E^D1^E^P^* heterozygous genotype (Table 3). This result indicates that the *E^D1^* allele is associated with a black coat phenotype and is inherited in a dominant pattern in crossbred BC pigs. According to the above results, we selected black boars and sows genotyped with *E^D1^E^D1^* from the F3 generation to reproduce offspring. As expected, all BC F4 cross pigs showed uniform black color in the whole production system.

### Reproductive performance of crossbred BC gilts compared with purebred CH gilts

Table 4 summarizes the reproductive performance of crossbred BC F4 gilts compared with purebred CH gilts. The mean number of teats was higher for BC gilts than for CH gilts (p< 0.001, 13.45 vs 12.14). The mean age at puberty of BC gilts was 168.44 d, although it was older than that of CH gilts (p<0.001, 125.45 d). The total number of pigs born (12.36 pigs) and number of pigs born alive (11.64 pigs) per litter were higher (p<0.001) for BC sows than for CH sows (10.31 pigs and 9.82 pigs, respectively). Breed effects were significant for litter birth weight and litter weaning weight. At birth and weaning at 28 d of age, litters from BC sows (11.92 kg and 65.87 kg, respectively) were heavier (p<0.001) than those from CH sows (8.33 kg and 49.40 kg, respectively).

### Growth and carcass attributes of crossbred BC pigs compared with purebred CH pigs

As expected, throughout the fattening period, the crossbred BC F4 pigs grew faster than the purebred CH pigs (p<0.001), with a higher average daily live weight gain (645.28 g vs 447.11 g); meanwhile, feed consumption was more efficient for BC crosses than for CH pigs (p<0.001, feed-to-gain ratio: 3.06 vs 4.03) (Table 5).

As shown in Table 5, the crossbred BC F4 pigs exhibited a superior carcass composition compared with those of purebred CH pigs. The slaughter weight was heavier for BC pigs at 211.70 d age than for CH pigs at 302.05 d of age (p<0.001, 112.56 kg vs 105.00 kg). The carcasses of BC pigs were longer than those of CH pigs (p<0.001, 83.35 cm vs 74.85 cm), and they had more ribs than the CH pigs (p<0.001, 14.50 vs 13.10). Importantly, the carcasses of BC pigs were more muscular than those of CH pigs (p<0.001), with a higher carcass lean meat composition (50.76% vs 42.58%), larger loin muscle area (32.61 cm^2^ vs 24.15 cm^2^), higher ham content (29.98% vs 25.03%), thinner back fat (26.44 mm vs 35.99 mm), and lower carcass fat content (23.65% vs 32.46%). Similar to CH pigs, BC pigs had thick skin (5.77 mm) and a high carcass skin rate (14.93%).

### Meat quality and muscle fatty acid composition of crossbred BC pigs compared with purebred CH pigs

Lick to CH pigs, crossbred BC F4 pigs displayed excellent meat quality attributes (Table 6). The meat from BC pigs showed ideal pH value (pH_45 min_ 6.32 and pH_24 h_ 5.90) and meat-color parameter (L_45 min_ 39.68 and L_24 h_ 42.41); meanwhile, the meat from BC pigs had strong water-holding capacity, with less water content (72.64%), very low drip loss (1.68%) and cooking loss (29.06%). Notably, the BC meat contained high intramuscular fat (IMF) content similar to CH pigs (3.72% vs 3.80%). In addition, the BC pigs displayed ideal muscle fiber parameters, with a small myofiber area (2,641.75 μm^2^), low shear force (6.16 kg) and firmness (26.59 kg/s).

More than 16 FAs were identified in the *longissimus dorsi* from both crossbred BC F4 pigs and purebred CH pigs, and the most prevalent FAs in all pigs were C18:1, C16:0, C18:0 and C18:2, accounting for more than 85% of all FAs (Table 7). The predominant saturated fatty acids (SFAs) were C16:0 and C18:0 in all pigs, while total concentrations of SFAs accounted for 34.14% in BC pigs and 49% in CH pigs. The predominant monounsaturated fatty acid (MUFA) in all pigs was C18:1 (52.48% for BC and 41.41% for CH). C18:2 was the main polyunsaturated fatty acid (PUFA) in all pigs, and the total concentrations of PUFA were significantly affected by breed, with BC pigs exhibiting a higher PUFA content than CH pigs (p<0.05, 14.03% vs 9.59%), which led to a PUFA:SFA ratio of 0.39 for BC crosses and 0.20 for CH pigs.

## DISCUSSION

### Black coat variation of pigs associated with the *MC1R* gene

Coat color is an important characteristic of various pig breeds and color variations may be useful in identifying the components of some specific crossbreeding schemes as well as contributing to the image associated with high-quality regional products [12]. In these crossbreeding experiments between Chinese indigenous CH pigs (uniform black) and imported BK pigs (domino black spotting) yielded a “dominant black” coat color hereditary pattern. A similar result was reported in which the allelism between the “uniform black type” and “domino black spotting type” may also be inferred from Large Black×BK crossed pigs [13].

Our observed segregation results led to the important discovery that the coat color variation of crossbred BC pigs is determined by the single *MC1R* gene, although more than eight color loci have been determined to be involved [14]. Twelve mutations were screened for the *MC1R* gene in BC crosses, which represent two typical *E^D1^* and *E^P^* alleles inferred according to the results of a previous report [11]. Our results indicate that the *E^D1^* allele associated with a black coat phenotype is inherited in a dominant pattern in crossbred BC pigs. Consequently, we selected black boars and gilts genotyped with homozygous *E^D1^E^D1^* from the F3 progeny and largely succeeded in producing the BC breed standard of black coat.

### Crossbreeding improves sow reproductive performance

The level of sow productivity is one of the most important production traits affecting the efficiency of a swine enterprise [15]. Crossbreeding programs have been extensively used to improve reproduction by exploiting breed additive effects, breed maternal effects, and heterosis. Young [16] reported that Chinese indigenous breeds Meishan, Fengjing, and Minzhu pigs can be used to produce crossbred gilts that have a higher level of reproductive performance than Duroc crossbred gilts. In this study, we found that crossbred BC F4 gilts had a higher litter and tear size, and the BC gilts are heavier at farrowing and at weaning than purebred CH gilts. Notably, the mean of 12.36 pigs for total litter size and 11.64 pigs for alive litter size of BC sows offers an advantage in litter size during the breeding process. A similar result was reported in which the cross sows from Chinese native Meishan, Fengjing, and Minzhu pigs showed a total number of pigs born (12.0 to 11.0 pigs) and a number of pigs born alive (11.3 to 10.7 pigs) per litter [14].

A favorable mean age at puberty of 168 d for crossbred BC gilts was found although BC gilts reached puberty later than CH gilts. A similar result was reported for a mean age at puberty of 118 and 217 d for purebred Meishan and its crossbred gilts [17]. However, purebred Duroc pigs averaged 234 d at puberty, compared with 210, 205, and 201 d for Hampshire, Pietrain, and Spot pigs [18].

### Crossbreeding improves growth performance and carcass composition

Previous reports revealed that the growth performance of hybrid pigs from Duroc×Dahe, Celta×Landrace, Celta×Duroc, and Duroc×Yanan was substantially improved compared with that of native pig breeds [3,4,6]. In our study, two important growth traits, weight gain and feed efficiency, were considerably improved in crossbred BC F4 pigs, indicating that the BC pigs reached a competitive slaughter age (approximately 180 d) at above 100 kg slaughter weight.

Meanwhile, crossbred BC pigs exhibited improved carcass characteristics, such as a moderate lean meat ratio (~50%) and backfat thickness (~2.6 cm). This result is similar to previous studies [3,4,6], which reported that the carcass characteristics of hybrid pigs were greatly improved compared with those of the native pig breeds and a mean lean meat ratio of 51% to 55% was found in the crosses from Duroc ×Dahe, Celta×Landrace, Celta×Duroc, and Duroc×Yanan. According to market demand for black pork in China, we suggest that it is perfectly suitable for black breeds to reach a mean of 53% to 55% for lean meat ratio (approximately 3% to 5% increase). Therefore, to achieve an ideal lean meat ratio, we will select back fat further down to 20 mm for alive back fat thickness at 180 d of age in the subsequent breeding process of BC pigs.

### Breed affecting meat quality characteristics

Meat quality is a key factor affecting how pork can be utilized. When choosing the best crossbreeding strategy, it is important to recognize pig breeds that determine meat quality attributes [19]. In this study, the crossbred BC and purebred CH pigs produced excellent meat-quality characteristics, which showed normal and high pH values compared to the recommended normal levels [20] (pH_45 min_ >6.1 and pH_24 h_ 5.5 to 6.0), normal and low meat color parameters according to NPPC standards (Minolta L-value levels of 37 to 49) [21], lower drip loss than those for foreign breeds above 3% [22], and smaller muscle-fiber areas than foreign hybrid pigs above 5,000 μm^2^ [19].

As the single most important parameter of meat quality, the IMF content is related to the organoleptic characteristics of pig meat and influences meat and meat-product quality [23]. An IMF content of 2% to 3% is suggested to be optimal for food quality [24,25]. Interestingly, the crossbred BC pigs in the present study exhibited relatively high IMF content (3.72%). Meanwhile, a higher PUFA:SFA ratio of IMF leads to better digestion rates and an improved digestibility of SFAs with emulsifying agents [26,27] and the recommended PUFA:SFA ratio is more than 0.4 [28]. Here, a similar PUFA: SFA ratio of ~0.39 was found in the BC pigs. BC pig meat with a high IMF content and PUFA:SFA ratio can meet the demand for high-quality niche pork products.

The superior meat quality properties for the crossbred BC pigs may be attributed to the breed attributes of their parents. Previous studies found that the BK sire pigs are superior for loin meat and eating [22], and these characteristics are, consequently, thought to be attributed to their higher overall likeability score [29,30] and improved acceptability compared with European commercial pork breeds [31]. Meanwhile, our results and those of a previous study indicate that CH pigs are also characterized by superior meat quality traits [1].

## CONCLUSION

The coat color of Berkshire×Chenghua (BC) cross pigs exhibits a “dominant black” phenotypic hereditary pattern and the new crossbred BC F4 pigs exhibit a uniform black coat pattern through proper selection of the sire and maternal pigs with the *E^D1^E^D1^* homozygous genotype for the *MC1R* gene. Meanwhile, the crossbred BC F4 pigs have an outstanding overall production performance, which shows that BC pigs have a relatively good maternal reproductive performance, market-competitive improved growth and carcass characteristics, and super meat-quality attributes. These results indicate that the new crossbred BC black pigs can be extensively used in commercial pig production to provide high-quality niche products.

## Figures and Tables

**Table 1:** Primer sequences and amplification conditions for the *MC1R* gene

Gene	Primer	Primer sequence	Binding region	Product size (bp)	Annealing temperature (°C)
*MC1R*	M-F1	GCTGAGCACAGGCGAGGTT	5′UTR	884	61
	M-R1	GGAAGCAGAGGCTGGACACC	Exon1		
	M-F2	CATCGCCAAGAACCGCAACC	Exon1	903	61
	M-R2	GGTCCAGCGTCCATACCTTCA	3′UTR		

*MC1R*, melanocortin 1 receptor; UTR, untranslated regions.

**Table 2:** Observation of coat color variation in crossbred BC^1)^ pigs

Progeny	Boar		Sow		Piglets		
		
	Breed	Coat color	Breed	Coat color	Coat color		Ratio of black: domino
F1^2)^	BK^1)^ (10)	Black	CH^1)^ (320)	Black	Black (3140)	Domino (0)	-
F2	F1 (30)	Black	F1 (588)	Black	Black (4434)	Domino (1472)	3:1
F3	F2 (9)	Black	F2 (182)	Black	Black (2038)	Domino (3)	-
F3	F2 (17)	Black	F2 (330)	Black	Black (3088)	Domino (610)	5:1

1) BC, Berkshire×Chenghua; BK, Berkshire; CH, Chenghua.

2) F1, F2, and F3 means the BC cross pigs from first, second, and third generation, respectively.

“-” Means that the ratio cannot be calculated or very large because coat color pattern of (nearly) all pigs is black.

**Table 3:** Mutation sites of the *MC1R* gene in CH^1)^, BK^1)^, and crossbred BC^1)^ pigs

Breed	Coat color pattern	Genotype locus	Allele/genotype	Mutation locus											

				5′UTR					CDS						
	
				215	220	242	371	414	490	505	722	744	802	809	1338
CH, F2	Black	*E* *^D1^* *E* *^D1^*	Allele	A	G	C	G	C	A	-	A	C	C	G	A
			Genotype	AA	GG	CC	GG	CC	AA	-	AA	CC	CC	GG	AA
F1^2)^, F2^2)^	Black	*E* *^D1^* *E* *^P^*	Allele	A&G	A&G	C&T	A&G	C&T	A&G	-CC	A&G	C&T	C&T	A&G	A&G
			Genotype	AG	AG	CT	AG	CT	AG	-CC	AG	CT	CT	AG	AG
BK, F2	Domino black spotting	*E* *^P^* *E* *^P^*	Allele	G	A	T	A	T	G	-CC	G	T	T	A	G
			Genotype	GG	AA	TT	AA	TT	GG	-CC	GG	TT	TT	AA	GG

*MC1R*, melanocortin 1 receptor; 5′UTR, 5′-untranslated regions; CDS, coding sequence.

1) CH, Chenghua; BK, Berkshire; BC, Berkshire×Chenghua.

2) F1 and F2 mean the BC cross pigs from first and second generation, respectively.

GenBank accession number AY960624.

**Table 4:** Reproductive performance of crossbred BC F4 gilts compared with purebred CH gilts

Traits	Breeds						SEM	p-value

	BC^1)^ (n = 927)			CH^1)^ (n = 322)				

	Mean	SD	CV (%)	Mean	SD	CV (%)		
Teat number	13.45	1.21	9.00	12.14	0.87	7.17	0.03	^***^
Puberty age (d)	168.44	28.38	16.85	125.45	21.39	17.05	2.60	^***^
Total no. born	12.36	2.13	17.23	10.31	2.01	19.50	0.07	^***^
No. born alive	11.64	1.74	15.62	9.82	1.88	19.14	0.03	^***^
Litter birth wt (kg)	11.92	1.78	14.93	8.33	1.65	19.81	0.25	^***^
Litter weaning wt (kg)	65.87	11.79	17.90	49.40	10.50	21.26	0.64	^***^

SEM, standard error of the mean; SD, standard deviation; CV, coefficient of variation.

1) BC, Berkshire×Chenghua; CH, Chenghua.

*** p<0.001.

**Table 5:** Growth and carcass traits of crossbred BC F4 pigs compared with purebred CH pigs

Traits	Breeds						SEM	p-value

	BC^1)^ (n = 60 and 30)			CH^2)^ (n = 60 and 30)				
	
	Mean	SD	CV (%)	Mean	SD	CV (%)		
Daily live weight gain (g/d)	645.28	30.02	4.65	447.11	42.72	9.55	10.44	^***^
Feed-to-gain ratio (kg/kg)	3.06	0.18	5.88	4.03	0.36	8.93	0.09	^***^
Slaughter age (day)	211.70	4.07	1.92	302.05	4.82	1.60	1.41	^***^
Slaughter weight (kg)	112.56	2.64	2.35	105.00	7.18	6.84	1.71	^***^
Carcass length (cm)	83.35	1.80	2.16	74.85	3.38	4.52	0.86	^***^
Dressing percentage (%)	73.27	2.39	3.26	74.21	1.14	1.54	0.59	n.s. ^2)^
Back fat thickness (mm)	26.44	3.56	13.46	35.99	3.52	9.78	1.12	^**^
Loin muscle area (cm2)	32.61	6.24	19.14	24.15	3.99	16.52	1.66	^***^
Skin thickness (mm)	5.77	0.87	15.08	6.03	1.15	19.07	0.41	n.s.
Number of ribs	14.50	0.53	3.66	13.10	0.45	3.44	0.18	^***^
Ham (%)	29.98	1.64	5.47	25.03	1.39	5.55	0.48	^***^
Carcass lean (%)	50.76	2.95	5.81	42.58	2.39	5.61	0.85	^***^
Carcass fat (%)	23.65	2.54	10.74	32.46	2.49	7.67	0.92	^***^
Carcass skin (%)	14.93	1.02	6.83	14.66	1.68	11.46	0.54	n.s.
Carcass bone (%)	10.74	0.85	7.91	10.29	0.87	8.45	0.27	n.s.

SEM, standard error of the mean; SD, standard deviation; CV, coefficient of variation.

1) BC, Berkshire×Chenghua; CH, Chenghua.

2) n.s., not significant (p>0.05).

** p<0.01,

*** p<0.001.

**Table 6:** Meat quality traits of crossbred BC F4 pigs compared with purebred CH pigs

Traits	Breeds						SEM	p-value

	BC^1)^ (n = 20)			CH^1)^ (n = 20)				
	
	Mean	SD	CV (%)	Mean	SD	CV (%)		
pH_45 min_	6.32	0.25	3.96	6.49	0.11	1.69	0.09	n.s.^2)^
pH_24 h_	5.90	0.25	4.24	5.95	0.23	3.87	0.11	n.s.
*L* (luminosity)_45 min_	39.68	2.06	5.19	40.23	2.58	6.41	0.74	n.s.
*L* (luminosity)_24 h_	42.41	3.32	7.83	42.35	3.77	8.90	1.12	n.s.
Crude protein content (%)	23.87	2.05	8.59	23.48	0.96	4.09	0.51	n.s.
Intramuscular fat content (%)	3.72	0.67	18.01	3.80	0.73	19.21	0.53	n.s.
Water content (%)	72.64	2.53	3.48	72.30	1.21	1.67	0.63	n.s.
Drip loss (%)	1.68	0.17	10.12	1.55	0.23	14.84	0.25	n.s.
Cooking loss (%)	29.06	2.32	7.98	28.87	2.42	8.38	0.75	n.s.
Shear force (kg)	6.16	0.45	7.31	9.37	0.39	4.16	0.97	^**^
Firmness (kg/s)	26.59	3.50	13.16	41.54	3.03	7.29	4.25	^**^
Myofibre area (μm^2^)	2,641.75	711.55	26.93	2,723.72	533.87	19.60	281.30	n.s.

SEM, standard error of the mean; SD, standard deviation; CV, coefficient of variation.

1) BC, Berkshire×Chenghua; CH, Chenghua.

2) n.s., not significant (p>0.05).

** p<0.01.

**Table 7:** Fatty acid composition of *M. longissimus lumborum* from crossbred BC F4 pigs compared with purebred CH pigs (% total fatty acids)

Traits	Breeds						SEM	p-value

	BC^1)^ (n = 20)			CH^1)^ (n = 20)				
	
	Mean	SD	CV (%)	Mean	SD	CV (%)		
C10:0	0.10	0.01	12.31	0.15	0.07	43.05	0.03	n.s.^2)^
C12:0	0.08	0.01	7.61	0.1	0.02	22.74	0.01	n.s.
C14:0	1.59	0.14	8.91	1.48	0.22	15.17	0.11	n.s.
C16:0	14.93	0.67	4.47	28.22	3.6	12.77	1.64	^***^
C16:1	4.01	0.22	5.43	3.93	1.02	26.06	0.47	n.s.
C17:0	0.15	0.02	14.91	0.21	0.04	16.87	0.02	^**^
C18:0	16.08	0.51	3.15	12.53	1.76	14.03	0.81	^***^
C18:1n9c	45.13	3.72	8.23	36.26	8.48	23.39	3.95	^*^
C18:2n6c	11.95	2.19	18.29	8.21	2.47	30.07	1.23	^**^
C18:3n3	0.27	0.04	15.32	0.25	0.04	15.69	0.02	n.s.
C20:0	0.25	0.04	14.28	0.29	0.07	23.71	0.03	n.s.
C20:2	0.39	0.07	18.14	0.61	0.24	39.76	0.11	n.s.
C20:3n3	0.44	0.08	19.01	0.27	0.09	34.25	0.01	n.s.
C21:0	0.81	0.05	6.47	4.05	6.59	162.72	1.11	n.s.
C22:1n9	3.33	0.69	20.61	0.03	0.01	24.77	0.16	^***^
C23:0	0.05	0.02	36.47	1.60	0.66	41.58	0.30	^***^
SFA	34.14	1.22	3.56	49.00	6.69	13.66	3.05	^***^
PUFA	14.03	11.39	81.19	9.59	2.84	29.67	1.41	^*^
MUFA	52.48	3.14	5.99	41.41	8.3	20.05	3.84	^**^
MUFA:SFA^3)^	1.54	0.14	8.81	0.85	0.23	27.55	0.11	^***^
PUFA:SFA^4)^	0.39	0.07	16.79	0.20	0.05	26.13	0.03	^***^

SEM, standard error of the mean; SD, standard deviation; CV, coefficient of variation; SFA, saturated fatty acid; PUFA, polyunsaturated fatty acid; MUFA, monounsaturated fatty acid.

1) BC, Berkshire×Chenghua; CH, Chenghua.

2) n.s., not significant (p>0.05).

3) MUFA:SFA, the ratio of MUFA to SFA;

4) PUFA:SFA, the ratio of PUFA to SFA.

* p<0.05;

** p<0.01;

*** p<0.001.
